# Oxidative DNA damage correlates with cell immortalization and mir-92 expression in hepatocellular carcinoma

**DOI:** 10.1186/1471-2407-12-177

**Published:** 2012-05-15

**Authors:** Cardin Romilda, Piciocchi Marika, Sinigaglia Alessandro, Lavezzo Enrico, Bortolami Marina, Kotsafti Andromachi, Cillo Umberto, Zanus Giacomo, Mescoli Claudia, Rugge Massimo, Farinati Fabio

**Affiliations:** 1 Department of Surgery, Oncology and Gastroenterology, Section of Gastroenterology, University of Padova, Via Giustiniani 2, Padova, 35128, Italy; 2 Department of Molecular Medicine, University of Padova, Via A. Gabelli 63, Padova, 35121, Italy; 3 Department of Medicine, University of Padova, Via A. Gabelli 61, Padova, 35121, Italy

**Keywords:** Hepatocellular carcinoma, 8-hydroxydeoxyguanosine, miR-92, Telomeric dysfunction, OGG1 gene

## Abstract

**Background:**

MicroRNAs expression has been extensively studied in hepatocellular carcinoma but little is known regarding the relationship, if any, with inflammation, production of reactive oxygen species (ROS), host’s repair mechanisms and cell immortalization. This study aimed at assessing the extent of oxidative DNA damage (8-hydroxydeoxyguanosine - 8-OHdG) in different phases of the carcinogenetic process, in relation to DNA repair gene polymorphism, telomeric dysfunction and to the expression of several microRNAs, non-coding genes involved in post-transcriptional regulation, cell proliferation, differentiation and death.

**Methods:**

Tissue samples obtained either at surgery, [neoplastic (HCC) and adjacent non-cancerous cirrhotic tissues (NCCT)] at percutaneous or laparoscopic biopsy (patients with HCV or HBV-related hepatitis or patients undergoing cholecystectomy) were analysed for 8-OHdG (HPLC-ED), OGG1 (a DNA repair gene) polymorphism (PCR-RFLP), telomerase activity, telomere length (T/S, by RT-PCR), Taqman microRNA assay and Bad/Bax mRNA (RT-PCR). Fifty-eight samples from 29 HCC patients (obtained in both neoplastic and peritumoral tissues), 22 from chronic hepatitis (CH) and 10 controls (cholecystectomy patients - CON) were examined.

**Results:**

Eight-OHdG levels were significantly higher in HCC and NCCT than in CH and CON (p=0.001). Telomerase activity was significantly higher in HCC than in the remaining subgroups (p=0.002); conversely T/S was significantly lower in HCC (p=0.05). MiR-199a-b, -195, -122, -92a and −145 were down-regulated in the majority of HCCs while miR-222 was up-regulated. A positive correlation was observed among 8-OHdG levels, disease stage, telomerase activity, OGG1 polymorphisms and ALT/GGT levels. In HCC, miR-92 expression correlated positively with telomerase activity, 8-OHdG levels and Bad/Bax mRNA.

**Conclusions:**

The above findings confirm the accumulation, in the progression of chronic liver damage to HCC, of a ROS-mediated oxidative DNA damage, and suggest that this correlates with induction of telomerase activity and, as a novel finding, with over-expression of miR-92, a microRNA that plays a role in both the apoptotic process and in cellular proliferation pathways.

## Background

Sound data suggest that reactive oxygen species (ROS) play a pathogenic role in carcinogenesis by inducing oxidative DNA damage, modulating gene expression, altering different signaling pathways and leading to a deregulation of cell proliferation and apoptosis [1-4]. ROS-induced DNA oxidation leads to a multitude of modifications to DNA bases, with 8-hydroxydeoxyguanosine (8-OHdG) representing the most frequent one. Eight-OHdG, a guanine adduct used as an index of DNA oxidative damage, induces a point mutation in the daughter DNA strands, accumulates in cell DNA and causes mispairing, thus demonstrating its mutagenic and potentially carcinogenic role [5]. During virus-related liver disease, both in humans and experimental models, an increased production of ROS has been documented, with a strong link between HCV core protein or HBV X protein and an oxidative “burst” [6,7]. These early events are followed in the progression of the disease by a build-up of genomic oxidative damage in patients with chronic hepatitis and cirrhosis, as documented in our own and other authors’ findings [8-11].

Several DNA repair mechanisms have specifically evolved and oxidized bases are repaired by a highly conserved base excision repair pathway, initiated by excision of the damaged base by glycosylases and by DNA strand cleavage. Four major DNA glycosylase, OGG1, NTH1, NEIL1 and NEIL2 have been characterized in human cells [12]. The OGG1 gene encodes for a DNA glycosilase/AP lyase which removes the oxidised DNA bases. It is located on chromosome 3p26.2 and a CG polymorphism at position 1245 exon 7 of the gene [with substitution of cysteine for serine at codon 326 (Ser326Cys)] has been described, that is associated with a significantly lower DNA repair activity by the coded enzyme [13].

Amongst the many potential targets of oxidative damage are microRNAs (miRNAs) [14]. miRNAs are a family of non-coding genes, involved in post-transcriptional gene regulation, in cell proliferation, differentiation, cell death and carcinogenesis, that have been reported to play an important role in chronic liver damage progression and hepatocellular carcinoma (HCC) development [15].

A list of miRNAs differentially expressed in HCC compared to non-cancerous liver has been indeed described [16], among which the liver specific miRNA, miR-122, often under-expressed in hepatic tumours, which also interacts with the 5′ noncoding region of HCV genome [17,18]. Several others miRNAs are involved in cell cycle control; some of these, such as the miR-17-92 cluster, miR-21, miR-221/miR-222*,* miR-224 and miR-146a, are up-regulated in HCC, others, including miR-125b, miR-1, miR-195, miR-223, miR-101 and miR-145, down-regulated [18,19]. These miRNAs may inhibit apoptosis, facilitate invasion and metastasis, act as either tumor suppressors or oncogenes [16] and have been associated with cell differentiation, self-renewal and tumor initiation *in vivo*, as in the case of the miR-181 family, overexpressed in hepatic cancer stem cells [20].

miRNAs, as oxidative DNA damage, are also involved in the regulation of telomerase activity [21], which is up-regulated in mutated cells activity [22]. Telomeric DNA indeed is particularly rich in guanine residues and, under ROS attack, is highly prone to 8-OHdG formation [23]. The resulting telomere shortening, with chromosome instability, involves the first phases of carcinogenesis while tumor progression is linked to a reactivation of telomerase activity, with telomere elongation and cell immortalization [24,25].

On these premises, the aims of this study were to:

confirm the accumulation of oxidative DNA damage in the different phases of the liver carcinogenetic process and ascertain whether OGG1 gene polymorphisms modulate the extent of damage;

evaluate, in the same model, telomerase activity, telomere length and any effect of 8-OHdG and of OGG1 gene polymorphism on both;

investigate the expression of a panel of miRNAs with possible direct or indirect interference on cellular oncogenes and tumor suppressors justifying their involvement in the pathogenesis of HCC, in relation to oxidative DNA damage, telomerase activity and factors involving in apoptotic mechanisms, including Bad and Bax [26].

## Methods

This study was carried out in tissue samples of patients with either HCC undergoing surgical resection or chronic virus-related liver damage undergoing US-guided liver biopsy and, as control group, in patients undergoing cholecystectomy.

Sixty-one patients (corresponding to 90 samples) entered the study;

twenty-nine patients with HCC, 20 males and 9 females, mean age 62 (±13), entered the study and during the surgical resection both a neoplastic sample (HCC) and a sample from the non-cancerous cirrhotic tissue surrounding the resected nodules (NCCT) were obtained, for a total of 58 samples. Etiology of the disease was as follows: HBV or HCV-related 15 patients (52%), alcohol 5 patients (17%), other factors or cryptogenic 9 patients (31%);

twenty-two patients with HCV or HBV-related chronic liver damage (CH) (20 HCV, 2 HBV) 12 males, 10 females, mean age 53 (±8), also entered the study. In this case, part of the liver biopsy samples (obtained by 16/17 Gauges Menghini modified needles under US guidance) was devoted to the planned studies while the bulk (always longer than 2 cm) was used for the routine histological examination;

finally, ten patients undergoing cholecystectomy were submitted, during laparoscopy, to liver biopsy with the same modalities as above (6 females and 4 males, mean age 56 (±7) (CON).

In each patient anti-HCV antibodies were looked for using a second-generation ELISA and all positive sera were confirmed by RIBA II assay. In all patients, anti-HCV sera positivity was confirmed by positive HCV-RNA levels determination using the Amplicor HCV test (Amplicor PCR Diagnostic, Hoffman-La Roche, Basel Switzerland). A standardized genotyping assay (Inno-Lipa HCV III, Innogenetics, Gent, Belgium) was used.

The HBV group consisted of individuals who were HBsAg, anti-HBe, and HBV-DNA positive at PCR. HBV serum markers were tested by radioimmunoassay (RIA) (Abbott, Chicago, IL, USA), while HBV-DNA levels were tested using a commercially-available fluid phase hybridization assay (Abbott, Chicago, IL, USA).

After obtaining informed consent, the patients provided full information relative to their drinking and smoking habits and completed a food frequency questionnaire with particular attention to their intake of vitamins. Patients with concurrent diseases or those taking medications capable of interfering with free radical production, such as non-steroidal anti-inflammatory drugs (NSAIDs) or anti-oxidants (vitamin C), were excluded from the study.

The study that was approved by the Ethic Committee of Padua Hospital.

### Genomic DNA extraction from biopsy and surgical liver sample

Genomic DNA extraction from surgical liver samples and biopsies was made on portions of tissues immediately snap-frozen in liquid nitrogen and stored at - 80°C until use. For DNA extraction we used a Wizard Genomic DNA Purification Kit (Promega Italia, Milano, Italy) according to the protocol provided. The liver tissues (approximately 10 mg) were homogenized with pestle in a solution of EDTA 0.5 M pH 8, Nuclei Lysis Solution and Proteinase K (20mg/ml).

### Quantification of 8-OHdG adduct

This assay was performed with a portion of the liver material (both surgical and biotic samples), and consisted of 3 steps: (*i*) genomic DNA extraction using a Wizard Genomic DNA Purification Kit (Promega Italia, Milano, Italy); (*ii*) nuclease P1 and alkaline phosphatase hydrolysis of DNA; (*iii*) 8-OHdG determination using HPLC-ED, which is a highly sensitive method with a detection limit of ~2 adducts per 10^5^ deoxyguanosine (dG). Following nuclease P1 and alkaline phosphatase hydrolysis, samples were filtered through 0.22-mm nylon filters (Scientific Resources, Alfatech, Genova, Italy), and 20 μL DNA per sample was injected in the HPLC (Alliance 2695, Waters, Milano). Eight-OHdG and normal deoxynucleosides were separated in a 3-mm Supelcosil LC-18-DB analytical column (7.5 cm × 4.6 mm, Supelco, Bellefonte, PA) equipped with a 5-mm SupelguardTM LC-18-DB guard column cartridge.

The solvent system consisted of an isocratic mixture of 90% of 50 mM potassium phosphate, pH 5.5, and 10% methanol at a 1 ml/min flow rate.

The 8-OHdG was detected using an electrochemical detector (ESA Coulochem II 5200 A, Bedford, MA) equipped with a high-sensitivity analytical cell, model 5011, with the oxidation potentials of electrodes 1 and 2 adjusted to 0.15 and 0.35 V, respectively. The levels of 8-OHdG were referred to the amount of dG detected in the same sample by UV absorbency at 254 nm. The amount of DNA was determined from a calibration curve *vs*. known amounts of calf thymus DNA. The 8-OHdG levels were expressed as the number of 8-OHdG adducts per 10^5^ dG. An 8-OHdG standard (Sigma-Aldrich, St. Louis, MO, USA), prepared immediately before the assay, was injected before any set of samples. The coefficient of variation was <10%; the amount of DNA required for the assay was 100 μg. Samples with lower amounts of DNA were rejected, since the risk of methodological error is only acceptable above this cut-off.

### OGG1 gene: PCR and Restriction Fragment Length Polymorphism (RFLP) Analyses

Cellular DNA isolated from tissue samples was analyzed by PCR to study the C:G transversion at nucleotide 1245 of the OGG1 gene. An aliquot of 100 ng of genomic DNA was added to a 25 μl PCR mixture and PCR was performed using the primers described by Kohno et al. [27], 5′-AGGGGAAGGTGCTTGGGGAA--3′ as the forward primer and 5′ –ACTGTCACTAGTCTCACCAG - 3′as the reverse primer.

The thermoprofile consisted of 30 cycles of denaturation at 94°C for 15 sec, annealing at 58°C for 15 sec and extension at 72°C for 40 sec, preceded by an initial denaturation step at 94°C for 2 min followed by a terminal extension at 72°C for 5 min. The 1245 C:G transversion was identified using PCR-RFLP. Briefly, 10 μl of the 200-bp PCR product was digested with Fnu4HI. The presence of a C:G transversion creates a Fnu4HI recognition site, which leads to digestion of the 200-bp PCR product into 2 fragments of 100 bp. Heterozygous subjects exhibit 2 fragments (200 and 100 bp), and a homozygous C:G transversion results in the appearance of a single fragment of 100 bp. Fnu4HI digests of PCR amplification products were observed by electrophoresis on 3% agarose gels after ethidium bromide staining.

### Protein extraction from surgical and bioptic liver sample

Liver samples were lysed in 200 μl of CHAPS buffer containing Protease Inhibitor Cocktail (Sigma-Aldrich, St. Louis, MO, USA) and incubated at 4°C for 30 min. The lysate was centrifuged at 14000 rpm for 45 min at 4°C and the supernatant was collected −80°C. The protein concentration was measured using RCDC Protein Assay (Bio-Rad, Milano, Italy).

### Real Time Quantitative–Telomeric Repeat Amplification Protocol (RTQ-TRAP) for telomerase activity

Each hepatic sample was assayed for telomerase activity, starting from protein extracts of the liver tissue stored at −80°C, by an ABI 7900 Sequence Detection System (Applied Biosystems, Foster City, CA, USA). The total volume of the reaction mixture was 25 μl in SYBR Green PCR Master Mix (Applied Biosystems, Foster City, CA, USA) contained DNA polymerase Ampli-Taq Gold, dNTPs and dUTP, 1X SYBR Green buffer, 0.2 μg of T_4_ gene protein, primers TS (5′-AATCCGTCGAGCAGAGTT-3′) 0.6 μM, ACX [5′-GCGCGG(CTTACC)_3_CTAACC-3′] 0.4 μM and 1 μg/μl of protein extract. The PCR was performed in a 96-well micro titer plate and the reaction mixture was first incubated at 25°C for 20 min to allow the telomerase in the protein extract to elongate the TS primer by adding TTAGGG repeat sequences.

PCR then started at 95°C for 10 min, followed by a 40-cycle amplification at 95°C 20 s and 60°C for 30 s. All samples were assayed in triplicate to test the reproducibility of the RTQ-TRAP assay, and in each assay also a protein extract of a cell line (PLC/PRF/5) as positive control and water as negative control were evaluated. Following amplification, a dissociation curve was performed in order to confirm the specificity of the reaction. Fluorescence signals and dissociation curves were collected and analyzed by ABI 7900 SDS 2.3 software so telomerase activity in cells line and samples was calculated based on the threshold cycle (Ct).

Telomerase activity in cells line and samples was extrapolated using a standard curve prepared with serial dilutions of Telomerase Control Oligo Standard (amol/μl) containing telomeric repeats (TAAGGG). Telomerase activity was expressed as percentage compared to that in PLC/PRF/5 cells.

### Telomere length analysis by quantitative PCR

Genomic DNA extracted was used also to measure telomere length by using method developed by Cawthon [28]. Two 96-well plates were prepared for each experiment, one of which containing telomere primers

(Tel-1:5′-GGTTTTTGAGGGTGAGGGTGAGGGTGAGGGTGAGGGT-3′, Tel-2: 5′- TCCCGACTATCCCTATCCCTATCCCTATCCCTATCCCTA-3′) and the other for 36B4, encoding acidic ribosomal phosphoprotein P0 used as the single copy gene (36B4u: 5′-CAGCAAGTGGGAAGGTGTAATCC-3′, 36b4d: 5′-CCCATTCTATCATCAACGGGTACAA-3′).

Each 25 μl PCR reaction included 40 ng of DNA, SYBR Green Master Mix (Applied Biosystems, Foster City, CA, USA) and primers at final concentrations 270 nM Tel-1 and 900 nM Tel-2 or 300 nM 36B4u and 500 nM 36B4d, respectively.

PCR amplification was performed in a ABI PRISM 7900 (Applied Biosystems, Foster City, CA, USA). In each plate, a standard curve was produced ranging from 4 to 80 ng of human reference DNA (Applied Biosystems, Foster City, CA, USA). The thermal cycling profile for the telomere amplification was 95°C for 10 min, followed by 30 cycles of 95°C for 5 s, 56°C for 10 s and 72°C for 60 s or 30 cycles of 95°C for 5 s, 58°C for 10 s and 72°C for 40 s. Following amplification, a dissociation curve was performed in order to confirm the specificity of the reaction.

All samples were assayed in triplicate to test the reproducibility and in each assay was evaluated a negative control. Fluorescence signals and dissociation curves were analyzed by ABI 7900 SDS 2.3 software. Telomeres/single copy gene (T/S) ratio for each sample were obtained by dividing the mean amount of telomeres by the mean amount of 36B4 gene.

### miRNAs expression: Taqman microRNA assay

We started from 0.1g of frozen tissue for each sample and RNA extraction was performed through mirVana miRNA isolation kit (Ambion, Austin, TX, USA), a method which combines phenol-chloroform and fiber-glass columns to obtain total RNA enriched in miRNAs. RNA was eluted in RNAse-free water and stored at −80°C; concentration and quality were checked with Nanodrop (Thermo Scientific, Canada, USA). The expression of a selected panel of miRNAs was then evaluated by mean of RT-PCR, using TaqMan microRNA assay (Applied Biosystems, Foster City, CA, USA). 10ng of RNA were reverse translated for each sample using looped primers specific for each miRNA and the obtained cDNA was assayed with real-time PCR with miRNA specific primers and TaqMan® probes. Data obtained for each miRNA were normalized versus a control RNA (RNU6B) and the relative expression for coupled tumour and normal samples was expressed by ΔΔCT method.

### Reverse transcription for Bad/Bax mRNA determination

For the synthesis of complementary DNA (cDNA), 2 μg of RNA were reverse transcribed in a final volume of 40 μl in the presence of 1X PCR buffer, 1 mM each of dNTPs (dATP, dTTP, dCTP, dGTP), 1 U RNase inhibitor, 2.5 μM random hexamers, and 2.5 U of murine leukemia virus (Perkin Elmer, Foster City, CA, USA). The reverse transcription reaction was completed at 25°C for 10 min, 42°C for 15 min and 99°C for 5 min, in a Perkin Elmer GeneAmp PCR System 2400. The cDNA was stored at −20°C.

### Quantitative absolute real-time PCR

Real-time PCR was conducted in an ABI 7900 Sequence Detection System (Applied Biosystems, Foster City, CA, USA) using SYBR Green I. The reaction was obtained on 96-well plates, in a 25 μL final volume containing 1X TaqMan buffer, 5.5 mmol of MgCl2, 200 μmol of nucleotides with dUTP, 0.25 U of AmpliTaq Gold Polymerase (SYBR Green Master Mix), 300 nM of each primer and 200 ng of cDNA template. Nucleotide sequences for the sense and antisense primers used for real-time PCR were: 5′-CTTTTGCTTCAGGGTTTCATCC-3′, 5′-TTGAGACACTCGCTCAGCTTCT-3′ for Bax [ENST00000356483], and the length of this amplicon was 119 bp; 5′- TCTATGCAAGTTTTGCCCTTTGTA-3′, 5′-GCCAGCCTGAATGAAATGA- 3′ for BI-1 [ENST00000267115], and the length of this amplicon was 84 bp; 5′-CCTGGCACCCAGCACAA- 3′, 5′-GCCGATCCACACGGAGTACT for β-actin [ENST00000158302], and the length of this amplicon was 70 bp. After one 2-min step at 50°C and a second step at 95°C for 10 min, samples underwent 45 cycles of 45 s at 94°C and then: 45 s at 60°C for Bax and β-actin; 45 s at 65°C for Bad. A final extension step was performed at 60°C for 10 min.

### Statistics

The data obtained were initially examined for their distribution with the Kolmogorov-Smirnov test and then compared by either Anova One Way or Kruskal Wallis test. Correlation analysis were performed by either linear correlation analysis or Spearman Rank correlation analysis, as appropriate. The chi square test was used when indicated. A p value < 0.05 was considered as significant. The StatsDirect and PASW Statistics programs were used.

The power analysis for the data evaluated with unpaired two sample Student t tests showed the all the comparison with controls, with an 80% power and a 5% α error, were based on numbers of patients large enough to allow comparison.

## Results

### 8-OHdG levels

**Figure 1:**
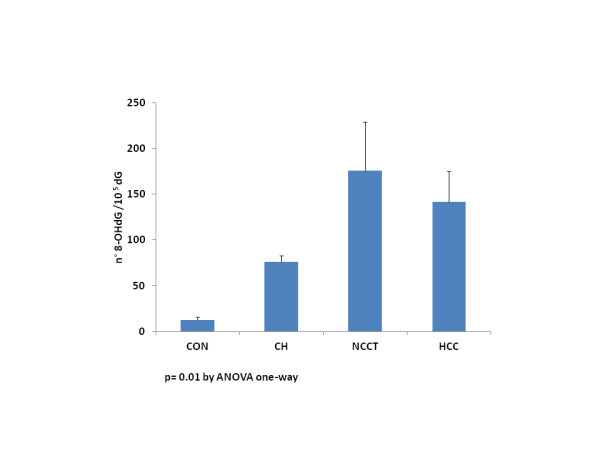
**8-OHdG levels in liver tissues in different stages of the disease progression.** 8-OHdG mean levels were significantly higher in NCCT than in other groups (CON, CH, HCC), p = 0.01 Anova one-way. *CON* Controls (10), *CH* HCV and HBV Positive Chronic Hepatites Tissues (22), *NCCT* Non-Cancerous Cirrhotic Tissues (29), *HCC* Neoplastic Tissues (29).

Oxidative DNA damage, as measured by the quantification of liver tissue 8-OHdG levels, was significantly higher in NCCT than in other groups (176±53 vs 142±33 HCC vs 76±7 CH vs 12±4 CON 8-OHdG/10^5^ dG, p=0.01, Anova one-way), also if no significant difference emerged between HCC and NCCT (Figure 1). With the limitations given by the relatively small sample size, no difference was observed between virus-related and non virus-related HCC in both HCC and NCCT tissues (data not shown).

### OGG1 gene polymorphism analysis

With respect to OGG1 gene polymorphism, heterozygous CG or homozygous GG polymorphisms were detected more frequently in CON and CH patients than in HCC, even not significantly so (50% vs 54% vs 26%, p=n.s).

### Telomerase activity

**Figure 2:**
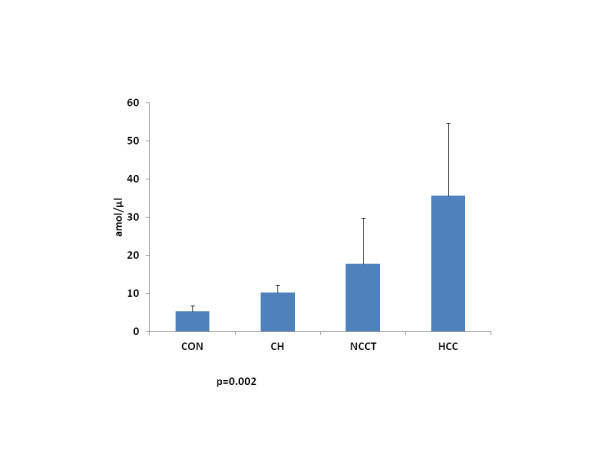
**Telomerase activity detected in liver tissues in different stages of the disease.** Mean/median levels of telomerase activity were higher in HCC patients’ group than other groups (p = 0.002 Kruskal-Wallis). *CON* controls (10), *CH* HCV- and HBV- Related Chronic Hepatitis Tissues (22), *NCCT* Non-Cancerous Cirrhotic Tissues (29), *HCC* Neoplastic Tissues (29).

As expected, an increased telomerase activity was detected in HCC (median 10.9 amol/μl, 0.6-77.9, 95% CI), versus all other patients groups (CON, CH and NCCT) (2.6 amol/μl, 1.7-8.6 95% CI; 6 amol/μl, 5.4-19 95% CI; 2.2 amol/μl, 0.1-22,1 95% CI respectively, p=0.002, Kruskal-Wallis) (Figure 2). Telomerase activity was higher than the upper 95% CI of CON in 34% of CH, 22% of NCCT and 53% of HCC.

No correlation was found between telomerase activity and OGG1 polymorphisms.

### Telomeres’ lenght

**Figure 3:**
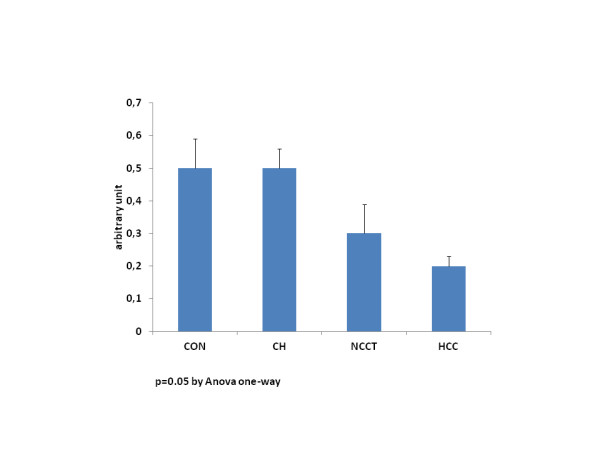
**Telomeres’ length detected in liver tissues in different stages of the disease.** Mean levels showed a significant reduction in the progression of disease to HCC (p = 0.05 Anova one-way). *CON* Controls (10), *CH* HCV and HBV Positive Chronic Hepatites Tissues (22), *NCCT* Non-Cancerous Cirrhotic Tissues (29), *HCC* Neoplastic Tissues (29).

Conversely, telomere length significantly decreased from CON and CH to NCCT and HCC (0.5±0.09 vs 0.5±0.06 vs 0.3±0.09 vs 0.2±0.03, p=0.05 Anova one-way) (Figure 3).

### miRNAs expression analysis and correlations

**Figure 4:**
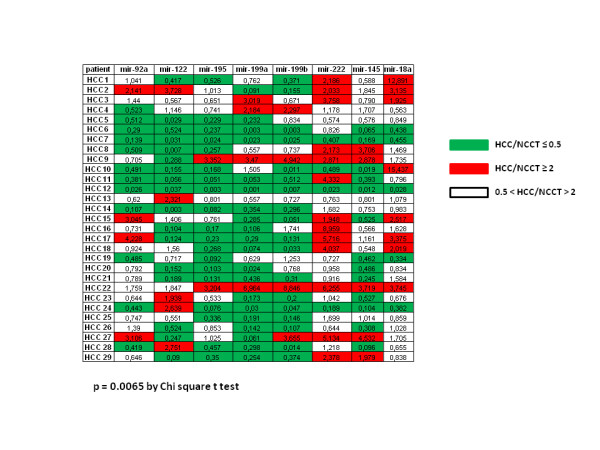
**Results of the miRNAs expression in the samples of liver tissues.** Each value represents the ratio of expression HCC/NCCT calculated using the ΔΔCt method (HCC/NCCT= 2^-^^ΔΔCt^ ) on the average of the repetitions of each sample. The inclusion in the expression categories has the ratio 2 (or 0.5) as threshold with a margin of 5% confidence. MiR-222 was more frequently hyperexpressed than any other miRNAs (p=0.0065). *NCCT* Non-Cancerous Cirrhotic Tissues (29), *HCC* Neoplastic Tissues (29).

The study of the levels of expression of the panel of miRNAs assayed with RT-PCR in coupled tumor and adjacent tissues and the correlation studies lead to the following results:

seven miRNAs showed a relevant deregulation in HCC tissues with respect to NCCT: miR-222 showed a high rate of over-expression (two-fold increase in 45% of the patients, significantly more frequently hyperexpressed than any other miRNAs, p=0.0065), while the levels of miR-92a, miR-122, miR-195, miR-199a, miR-199b and miR-145 showed a lower expression in HCC tissues (two-fold decrease ranging from 41% of patients for miR-92a up to 69% of patients for mir-199a). MiR-18a expression was not different in the two tissues (Figure 4).

**Table 1:** Multiple and linear correlation analysis

	**8-OHdG**
Histological diagnosis	p = 0.01
NCCT telomerase activity	p = 0.0001
HCC OGG1 polymorphism	p = 0.05
ALT	p = 0.01
GGT	p = 0.03
	**miR-92**
HCC 8-OHdG	p = 0.05
HCC telomerase activity	p = 0.004
HCC Bax mRNA	p = 0.05
NCCT Bax mRNA	p = 0.05
	**8-OHdG**
HCC Bad mRNA	p = 0.05

*NCCT* Non-Cancerous Cirrhotic Tissues (29)

*HCC* Neoplastic Tissues (29)

## Discussion

This study was aimed at defining the interrelationships among oxidative damage, telomerase activity, telomeric dysfunction and miRNAs expression in the progression of chronic liver damage and HCC development.

Our data confirm what ourselves and other authors already published on the relevance of oxidative DNA damage in the progression from chronic liver damage to HCC in virus-related liver damage, confirming the progressive accumulation of DNA damage that reaches a maximum in both the neoplastic and in the adjacent non-cancerous cirrhotic tissues. This last finding clearly indicates the presence of a “field defect” that most probably contributes to the multifocal and relapsing nature of HCC in cirrhosis [29].

Our results demonstrated the lack of a specific significant association of OGG1 polymorphisms with HCC development. CG and/or GG polymorphisms, which are reportedly linked to a lower repair capacity in other cancers [30,31], as also confirmed in our own experience [32], were less frequently detected in cirrhosis and HCC samples. There are several other repair enzymes potentially involved in repairing DNA oxidative damage [33] and the lack of association with OGG1 polymorphisms probably indicates that, in the liver, other repair mechanisms are more relevant than OGG1. On the other hand, a significant correlation was documented between OGG1 polymorphisms and 8-OHdG levels, overall, thus confirming that, when the polymorphism associated with lower repair capacity is present, oxidative DNA damage accumulates, irrespective of the phase of the disease and of the activity of other repair systems.

Our findings also confirm that an increased telomerase activity is a feature of HCC, in agreement with the data reporting that a high percentage of HCC cases show an increased telomerase activity [34-36]. Using the data obtained in the control population, telomerase activity was increased in over 50% of the cases. Despite this increased activity, telomeres were significantly shorter in HCC than observed in chronic hepatitis. Other authors reported that HCC tissues are frequently characterized by an increased oxidative damage which contributes to the acceleration of telomere shortening and the activation of telomerase in cancer cells. Telomeres act as protective caps at the ends of chromosomes and telomere shortening promotes chromosomal instability. It is not therefore surprising that telomere shortening occurs during the course of chronic liver disease and in hepatocarcinogenesis.

The data we obtained with respect to miRNAs in the study are largely in agreement with well established literature reports on miRNAs expression in HCC. In particular, miR-222 was found overexpressed in the neoplastic tissue, consistently with other findings on HCC, pancreatic and gastric cancer [18]. Also the under expression we observed with respect to miR-199a, miR-199b, miR-195 and miR-122 in tumour samples supports what reported by many other groups in HCC [37,38] as well as in other cancers [39-41]. MiR-92a, part of miR-17-92 cluster, was instead found under-expressed in nearly one half of the tumour samples investigated [42]. This result is in contrast with what reported in several publications regarding an overexpression of the entire miR-17-92 cluster in most cancers. In many cases however the individual behaviour of miR-92a was not well defined in these papers [43,44]. On the other hand, our data are in agreement with a number of studies which reported a decrease in miR-92a expression in association with a deletion in chromosome 13q that encodes for the miR-17-92 cluster [45,46], a relatively frequent finding in cancer samples.

The miR-17-92 cluster indeed encodes seven miRNAs, which are tightly grouped within an 800 base-pair region of chromosome 13q and apparently play a role in both the apoptotic process and in cellular proliferation pathways. In fact, some members of the E2F family of transcription factors, which are critical regulators of cell cycle and apoptosis (by inducing the expression of genes that drive progression from G_1_ into S phase), were among the first experimentally verified target of the miR-17-92 cluster [47]. Nevertheless, the way by which miR-92a deregulation can influence carcinogenesis in virus-related liver still needs to be elucidated and some hypotheses have been formulated to explain the mechanisms behind this finding:

a. the down regulation of miR-17-92 family may be advantageous for cancer development: as previously mentioned, loss-of-heterozygosis at miR-17-92 locus (13q31.3) has been observed in multiple tumour types (including HCC) [45]; moreover, the down regulation of another member of the cluster, miR-17, has been reported to contribute to breast cancer development through over expression of its target, the oncogene AIB1 [48].

b. many of the miRNAs that have been reported to be up regulated in a variety of cancer cells, as miR-17-92, show a lower expression associated with in vitro stress conditions [49,50]. For example, the induction of ROS by mean of oxidant agents has been shown to reduce the expression of a group of miRNAs, including members of this cluster: miR-17, miR-18a, miR-20a, miR-92a and the paralogs miR-106a and miR-20b [14].

c. As shown by our results, miR-92 may also participate in the complex interplay of factors up or down-regulating apoptosis [51].

## Conclusions

Our data indicate the existence of a link between oxidative damage and miR-92 expression. In particular, this study demonstrates, for the first time and in vivo, that down-regulation of miR-92 significantly correlates with both the extent of oxidative DNA damage and telomerase activity in HCC. Additionally, miR-92 levels also correlated with Bad and Bax mRNA, involved in apoptotic mechanisms.

Overall, our findings suggest that in chronic HCV-related liver damage, the persistently increased ROS production leads to accumulation of oxidative DNA damage, which is only partially repaired by OGG1 in association with other repair mechanisms. A deranged expression of several miRNAs is confirmed, one of which (miR-92) is apparently correlated with the extent of oxidative stress and with telomerase activation in HCC tissues.

## Abbreviations

ROS, Reactive Oxygen Species; 8-OHdG, 8-hydroxydeoxyguanosine, miRNAs, MicroRNAs; HCC, Hepatocellular carcinoma; NCCT, non-cancerous cirrhotic tissue surrounding the resected nodules; CH, HCV or HBV-related chronic liver damage; RFLP, Restriction fragment length polymorphism; RTQ-TRAP, Real time quantitative–telomeric repeat amplification protocol; T/S, Telomeres/single copy gene ratio; dG, Deoxyguanosine.

## Competing interests

The authors declare that they have no competing interests.

## Authors’ contributions

CR performed the majority of experiments and wrote the manuscript; PM, SA, LE, BM and KA performed the experiments; CU and ZG provided the surgical liver samples; MC and RM performed the histological analysis; FF designed the study and controlled the report. All authors read and approved the final manuscript.

## Funding

The study was supported in part by Ricerca Scientifica 2009 (cod: 60A07-9227/09), Padua University.

## Pre-publication history

The pre-publication history for this paper can be accessed here:

http://www.biomedcentral.com/1471-2407/12/177/prepub

## References

[B1] Marx J Inflammation and cancer: the link grows stronger Science 2004 306 966 968 10.1126/science.306.5698.966 15528423

[B2] Klaunig JE Kamendulis LM Hocevar BA Oxidative stress and oxidative damage in carcinogenesis Toxicol Pathol 2010 38 96 109 10.1177/0192623309356453 20019356

[B3] Bartsch H Nair J Chronic inflammation and oxidative stress in the genesis and perpetuation of cancer: role of lipid peroxidation, DNA damage and repair Langenbecks Arch Surg 2006 391 499 510 10.1007/s00423-006-0073-1 16909291

[B4] Liou GY Storz P Reactive oxygen species in cancer Free Radical Res 2010 44 479 496 10.3109/10715761003667554 20370557 PMC3880197

[B5] Kuchino Y Mori F Kasai H Inoue H Iwai S Miura K Ohtsuka E Nishimura S Misreading of DNA templates containing 8-hydroxydeoxyguanosine at the modified base and at adjacent residues Nature 1987 327 77 79 10.1038/327077a0 3574469

[B6] Sasaki Y Does oxidative stress participate in the development of hepatocellular carcinoma? J Gastroenterol 2006 41 1135 1148 17287893 10.1007/s00535-006-1982-z

[B7] Tsai WL Chung RT Viral hepatocarcinogenesis Oncogene 2010 29 2309 2324 10.1038/onc.2010.36 20228847 PMC3148694

[B8] Farinati F Cardin R Bortolami M Guido M Rugge M Oxidative damage, pro-inflammatory cytokines, TGF-alpha and c-myc in chronic HCV-related hepatitis and cirrhosis World J Gastroenterol 2006 12 2065 2069 16610058 10.3748/wjg.v12.i13.2065 PMC4087686

[B9] Farinati F Cardin R Bortolami M Burra P Russo FP Rugge M Guido M Sergio A Naccarato R Hepatitis C virus: from oxygen free radicals to hepatocellular carcinoma J Viral Hepat 2007 14 821 829 18070284 10.1111/j.1365-2893.2007.00878.x

[B10] Fujita N Horiike S Sugimoto R Tanaka H Iwasa M Kobayashi Y Hasegawa K Ma N Kawanishi S Adachi Y Kaito M Hepatic oxidative DNA damage correlates with iron overload in chronic hepatitis C patients Free Rad Biol Med 2007 42 353 362 10.1016/j.freeradbiomed.2006.11.001 17210448

[B11] Fujita N Sugimoto R Ma N Tanaka H Iwasa M Kobayashi Y Kawanishi S Watanabe S Kaito M Takei Y Comparison of hepatic oxidative DNA damage in patients with chronic hepatitis B and C J Viral Hepat 2008 15 498 507 10.1111/j.1365-2893.2008.00972.x 18331251

[B12] Ide H Kotera M Human DNA glycosylases involved in the repair of oxidatively damaged DNA Biol Pharm Bull 2004 27 480 485 10.1248/bpb.27.480 15056851

[B13] Shinmura K Yokota J The OGG1 gene encodes a repair enzyme for oxidatively damaged DNA and is involved in human carcinogenesis Antioxid Redox Signal 2001 3 597 609 10.1089/15230860152542952 11554447

[B14] Wang Z Liu Y Han N Chen X Yu W Zhang W Zou F Profiles of oxidative stress-related microRNA and mRNA expression in auditory cells Brain Res 2010 1346 14 25 20510889 10.1016/j.brainres.2010.05.059

[B15] Ladeiro Y Couchy G Balabaud C Bioulac-Sage P Pelletier L Rebouissou S Zucman-Rossi J MicroRNA profiling in hepatocellular tumors is associated with clinical features and oncogene/tumor suppressor gene mutations Hepatology 2008 47 1955 1963 10.1002/hep.22256 18433021

[B16] Braconi C Patel T MicroRNA expression profiling: a molecular tool for defining the phenotype of hepatocellular tumors Hepatology 2008 47 1807 1809 10.1002/hep.22326 18506877

[B17] Gramantieri L Ferracin M Fornari F Veronese A Sabbioni S Liu CG Calin GA Ferrazzi E Grazi GL Croce CM Bolondi L Negrini M Cyclin G1 is a target of miR-122a, a microRNA frequently down-regulated in human hepatocellular carcinoma Cancer Res 2007 67 6092 6099 10.1158/0008-5472.CAN-06-4607 17616664

[B18] Gramantieri L Fornari F Callegari E Sabbioni S Lanza G Croce CM Bolondi L Negrini M MicroRNA involvment in hepatocellular carcinoma J Cell Mol Med 2008 12 2104 2189 10.1111/j.1582-4934.2008.00533.x PMC4514099 19120703

[B19] Xu T Zhu Y Xiong Y Ge YY Yun JP Zhuang SM MicroRNA-195 suppresses tumorigenicity and regulates G1/S transition of human hepatocellular carcinoma cells Hepatology 2009 50 113 121 10.1002/hep.22919 19441017

[B20] Ji J Yamashita T Budhu A Forgues M Jia HL Li C Deng C Wauthier E Reid LM Ye QH Qin LX Yang W Wang HY Tang ZY Croce CM Wang XW Identification of microRNA-181 by genome-wide screening as a critical player in EpCAM-positive hepatic cancer stem cells Hepatology 2009 50 472 480 10.1002/hep.22989 19585654 PMC2721019

[B21] Koziel JE Fox MJ Steding CE Sprouse AA Herbert BS Medical genetics and epigenetics of telomerase J Cell Mol Med 2011 15 457 467 10.1111/j.1582-4934.2011.01276.x 21323862 PMC3922369

[B22] Nishikawa T Nakajima T Katagishi T Okada Y Jo M Kagawa K Okanoue T Itoh Y Yoshikawa T Oxidative stress may enhance the malignant potential of human hepatocellular carcinoma by telomerase activation Liver Int 2009 29 846 856 10.1111/j.1478-3231.2008.01963.x 19141026

[B23] Oikawa S Kawanishi S Site-specific DNA damage at GGG sequence by oxidative stress may accelerate telomere shortening FEBS Lett 1999 453 365 368 10.1016/S0014-5793(99)00748-6 10405177

[B24] Matusomi K Hahn WC Telomerase and tumorigenesis Cancer Lett 2003 194 163 172 10.1016/S0304-3835(02)00703-6 12757974

[B25] Artandi SE DePinho RA Telomeres and telomerase in cancer Carcinogenesis 2010 31 9 18 10.1093/carcin/bgp268 19887512 PMC3003493

[B26] Kotsafti A Farinati F Cardin R Burra P Bortolami M Bax inhibitor-1 down-regulation in the progression of chronic liver diseases BMC Gastroenterol 2010 10 35 42 10.1186/1471-230X-10-35 20359348 PMC2873598

[B27] Kohno T Shinmura K Tosaka M Tani M Kim SR Sugimura H Nohmi T Kasai H Yokota J Genetic polymorphisms and alternative splicing of the hOGG1 gene, that is involved in the repair of 8-hydroxyguanine in damaged DNA Oncogene 1998 16 3219 3225 10.1038/sj.onc.1201872 9681819

[B28] Cawthon RM Telomere measurement by quantitative PCR Nucleic Acids Res 2002 30 e47 10.1093/nar/30.10.e47 12000852 PMC115301

[B29] Jungst C Cheng B Gehrke R Schmitz V Nischalke HD Ramakers J Schramel P Schirmacher P Sauerbruch T Caselmann WH Oxidative DNA damage is increased in human liver tissue adjacent to hepatocellular carcinoma Hepatology 2004 39 1663 1672 10.1002/hep.20241 15185308

[B30] Obtulowicz T Swoboda M Speina E Gackowski D Rozalski R Siomek A Janik J Janowska B Ciesla JM Jawien A Banaszkiewicz Z Guz J Dziaman T Szpila A Olinski R Tudek B Oxidative stress and 8-oxoguanine repair are enhanced in colon adenoma and carcinoma patients Mutagenesis 2010 25 463 471 10.1093/mutage/geq028 20534734

[B31] Yuan W Xu L Feng Y Yang Y Chen W Wang J Pang D Li D The hOGG1 Ser326Cys polymorphism and breast cancer risk: a meta-analysis Breast Cancer Res Treat 2010 122 835 842 10.1007/s10549-009-0722-5 20058067

[B32] Farinati F Cardin R Bortolami M Nitti D Basso D de Bernard M Cassaro M Sergio A Rugge M Oxidative DNA damage in gastric cancer: CagA status and OGG1 gene polymorphism Int J Cancer 2008 123 51 55 10.1002/ijc.23473 18366059

[B33] Mitra S Boldogh I Izumi T Hazra TK Complexities of the DNA base excision repair pathway for repair of oxidative DNA damage Environ Mol Mutagen 2001 38 180 190 10.1002/em.1070 11746753 PMC4927302

[B34] Liu DY Peng ZH Qiu GQ Zhou CZ Expression of telomerase activity and oxidative stress in human hepatocellular carcinoma with cirrhosis World J Gastroenterol 2003 9 1859 1862 12918139 10.3748/wjg.v9.i8.1859 PMC4611562

[B35] Saini N Srinivasan R Chawla Y Sharma S Chakraborti A Rajwanshi A Telomerase activity, telomere length and human telomerase reverse transcriptase expression in hepatocellular carcinoma is independent of hepatitis virus status Liver Int 2009 29 1162 1170 10.1111/j.1478-3231.2009.02082.x 19627485

[B36] Ozturk M Arslan-Ergul A Bagislar S Senturk S Yuzugullu H Senescence and immortality in hepatocellular carcinoma Cancer Lett 2009 286 103 113 10.1016/j.canlet.2008.10.048 19070423

[B37] Jiang J Gusev Y Aderca I Mettler TA Nagorney DM Brackett DJ Roberts LR Schmittgen TD Association of MicroRNA Expression in Hepatocellular Carcinomas with Hepatitis Infection, Cirrhosis and Patient Survival Clin Cancer Res 2008 14 419 427 10.1158/1078-0432.CCR-07-0523 18223217 PMC2755230

[B38] Budhu A Jia H-L Forgues M Liu C-G Goldstein D Lam A Zanetti KA Ye Q-H Qin L-X Croce CM Tang Z-Y Wang XW Identification of Metastasis- Related MicroRNAs in Hepatocellular Carcinoma Hepatology 2008 47 897 907 10.1002/hep.22160 18176954

[B39] Nam EJ Yoon H Kim SW Kim H Kim YT Kim JH Kim JW Kim S MicroRNA expression profiles in serous ovarian carcinoma Clin Cancer Res 2008 14 2690 2695 10.1158/1078-0432.CCR-07-1731 18451233

[B40] Ichimi T Enokida H Okuno Y Kunimoto R Chiyomaru T Kawamoto K Kawahara K Toki K Kawakami K Nishiyama K Tsujimoto G Nakagawa M Seki N Identification of novel microRNA targets based on microRNA signatures in bladder cancer Int J Cancer 2009 125 345 352 10.1002/ijc.24390 19378336

[B41] Song B Ju J Impact of miRNAs in gastrointestinal cancer diagnosis and prognosis Expert Rev Mol Med 2010 12 e33 20942990 10.1017/S1462399410001663

[B42] Petrocca F Vecchione A Croce CM Emerging Role of miR-106b-25/miR-17-92 Clusters in the Control of Transforming Growth Factor β Signaling Cancer Res 2008 68 8191 8194 10.1158/0008-5472.CAN-08-1768 18922889

[B43] Manni I Artuso S Careccia S Rizzo MG Baserga R Piaggio G Sacchi A The micro RNA miR-92 increases proliferation of myeloid cells and by targeting p63 modulates the abundance of its isoforms FASEB J 2009 23 3957 3966 10.1096/fj.09-131847 19608627

[B44] Garofalo M Croce CM MicroRNAs: Master Regulators as Potential Therapeutics in Cancer Annu Rev Pharmacol Toxicol 2011 51 25 43 10.1146/annurev-pharmtox-010510-100517 20809797

[B45] Lin YW Sheu JC Liu LY Chen CH Lee HS Huang GT Wang JT Lee PH Lu FJ Loss of heterozygosity at chromosome 13q in hepatocellular carcinoma: identification of three independent regions Eur J Cancer 1999 35 1730 1734 10.1016/S0959-8049(99)00205-1 10674021

[B46] Zhang L Huang J Yang N Greshock J Megraw MS Giannakakis A Liang S Naylor TL Barchetti A Ward MR Yao G Medina A O’brien-Jenkins A Katsaros D Hatzigeorgiou A Gimotty PA Weber BL Coukos G MicroRNAs exhibit high frequency genomic alterations in human cancer Proc Natl Acad Sci USA 2006 103 9136 9141 10.1073/pnas.0508889103 16754881 PMC1474008

[B47] Mendell JT Myriad roles for the miR-17-92 cluster in development and disease Cell 2008 133 217 222 10.1016/j.cell.2008.04.001 18423194 PMC2732113

[B48] Hossain A Kuo MT Saunders GF Mir-17-5p regulates breast cancer cell proliferation by inhibiting translation of AIB1 mRNA Mol Cell Biol 2006 26 8191 8201 10.1128/MCB.00242-06 16940181 PMC1636750

[B49] Li G Luna C Qiu J Epstein DL Gonzalez P Alteration in miRNA expression in stress-induced cellular senescence Mech Ageing Dev 2009 130 731 741 10.1016/j.mad.2009.09.002 19782699 PMC2795064

[B50] Babar IA Slack FJ Weidhaas JB miRNA modulation of the cellular stress response Future Oncol 2008 4 289 298 10.2217/14796694.4.2.289 18407740

[B51] Danial NN BAD: undertaker by night, candyman by day Oncogene 2009 27 S53 S70 19641507 10.1038/onc.2009.44

