# Correction: Oxidative DNA damage correlates with cell immortalization and mir-92 expression in hepatocellular carcinoma

**DOI:** 10.1186/1471-2407-14-284

**Published:** 2014-04-23

**Authors:** Romilda Cardin, Marika Piciocchi, Alessandro Sinigaglia, Enrico Lavezzo, Marina Bortolami, Andromachi Kotsafti, Umberto Cillo, Giacomo Zanus, Claudia Mescoli, Massimo Rugge, Fabio Farinati

**Affiliations:** 1 Department of Surgical and Gastroenterological Sciences, Section of Gastroenterology, Padua University, Padua, Italy; 2 Department of Histology, Microbiology and Medical Biotechnologies, Padua University, Padua, Italy; 3 Department of Surgical and Gastroenterological Sciences, Hepatobiliary Surgery and Liver Transplant Unit, Padua University, Padua, Italy; 4 IRCCS – Venetian Oncology Institute (IOV), Padua, Italy; 5 Department of Medical Diagnostic Sciences & Special Therapies, Pathology Unit, Padua University, Padua, Italy; 6 Dipartimento di Scienze Chirurgiche e Gastroenterologiche, Policlinico Universitario, Via Giustiniani 2, Padova 35128, Italy

## Correction

After publication of this work [1], we noted that all the names and surnames of the authors have been swapped. Now the order of names and surnames of all authors has been corrected as follows: “Cardin R, Piciocchi M, Sinigaglia A, Lavezzo E, Bortolami M, Kotsafti A, Cillo U, Zanus G, Mescoli C, Rugge M, Farinati F” (Surname Name).

## Pre-publication history

The pre-publication history for this paper can be accessed here:

http://www.biomedcentral.com/1471-2407/14/284/prepub
